# Mycological Methods for Routine Air Sampling and Interpretation of Results in Operating Theaters

**DOI:** 10.3390/diagnostics14030288

**Published:** 2024-01-29

**Authors:** Rok Tomazin, Tadeja Matos

**Affiliations:** Institute of Microbiology and Immunology, Faculty of Medicine, University of Ljubljana, 1000 Ljubljana, Slovenia; rok.tomazin@mf.uni-lj.si

**Keywords:** filamentous fungi, settle plate method, active sampling methods, healthcare-associated infections, mycoses

## Abstract

Many infectious diseases are transmitted via the air and are, therefore, particularly difficult to combat. These infections include various invasive mycoses caused by molds. The usual route of infection is the inhalation of conidia. In hospitals, infection can also occur through the deposition of conidia in otherwise sterile anatomical sites during surgical and other invasive procedures. Therefore, knowledge of airborne mold concentrations can lead to measures to protect patients from fungal infections. The literature on this topic contains insufficient and sometimes ambiguous information. This is evidenced by the fact that there are no international recommendations or guidelines defining the methodology of air sampling and the interpretation of the results obtained. Surgical departments, intensive care units and medical mycology laboratories are, therefore, left to their own devices, leading to significant differences in the implementation of mycological surveillance in hospitals. The aim of this mini-review is to provide an overview of the current methods of air sampling and interpretation of results used in medical mycology laboratories.

## 1. Introduction

Fungal infections in healthcare settings occur in the context of contaminated infusion preparations, procedures on primarily sterile anatomical sites, transmission by the hands of healthcare personnel, biological material and airborne transmission [1,2,3,4]. Nosocomial invasive mycoses are a problem, especially in poorly equipped medical facilities [1]. Most of the cases described relate to infections with species from the genus *Aspergillus* and the order *Mucorales*, which are otherwise the most important and common causes of invasive mycoses due to molds [1,4,5]. Hospital-acquired aspergillosis and mucormycosis are mainly recorded in intensive care units, neonatology, hematology and transplant departments [4,5]. In these departments, there are patients who have the highest risk of developing invasive infections due to damaged anatomical barriers and/or immunocompromised conditions [4,5,6]. Infections are usually caused by the inhalation of conidia or sporangiospores and their colonization of exposed, primarily sterile, anatomical sites [4,5,6]. Nosocomial fungal infections of the lower respiratory tract, which can spread via the bloodstream to various organs and cause systemic infections with high mortality, are a common feature of most opportunistic molds and occur mainly in immunocompromised hospitalized patients [4,5]. In the hospital context, postoperative wound infections and primary skin infections as a result of the colonization of conidia/sporangiospores in the surgical wound or on damaged skin are also important [4,5]. Among the nosocomial mucormycoses, the gastrointestinal form is the most common, occurring mainly in premature infants and people after surgical interventions in the abdominal cavity [4]. Studies have consistently found the presence of fungi in the air of operating theatres, with *Aspergillus* being a common genus [7,8,9]. The level of biocontamination varies, with higher levels in working operating theatres [8,9]. The source of biocontamination is usually construction and renovation work near departments with high-risk patients or in the vicinity of hospitals [5]. Conidia and sporangiospores spread indoors through inadequate ventilation of hospital rooms (open windows, unfiltered air supply, inadequately cleaned filters, etc.) and through contaminated objects such as various syringes, needles, wooden tongue depressors, gauze, electronic devices in the operating theatres and many others [4,5,10]. The use of air-conditioning systems has been shown to reduce the number of microorganisms, including fungi, in the hospital environment [7]. However, the filters of air conditioning units can also be a source of fungal contamination [11]. These findings highlight the importance of preforming regular air sampling and maintenance of air-conditioning systems in operating theatres to minimize the risk of fungal infections [12,13,14]. The aim of this mini-review is to provide an overview of the current methods of air sampling and interpretation of results used in medical mycology laboratories.

## 2. Aeromycota—A Fungal Community in the Air

The source of nosocomial mycoses is the nosocomial aeromycota, a group of fungi, mainly spores and conidia, present in the air of a hospital. Several studies have been conducted that provide information on the diversity of aeromycota in different enclosed spaces, both in hospitals and in other public and private institutions [1,15,16]. Although the studies are very difficult to compare due to the use of different sampling methods, geographical location, season and some other aerobiological variables. *Cladosporium*, *Penicillium* and *Aspergillus* appear to be the most common fungal genera in enclosed spaces in all studies [1,15,16,17]. *Aspergillus* spp. is the only one of the three molds mentioned that can cause invasive infections. In addition to *Cladosporium* spp., *Penicillium* spp. and *Aspergillus* spp., some other opportunistic molds such as *Rhizopus* spp., *Alternaria* spp. and *Trichoderma* spp. as well as mycotoxin-releasing molds such as *Aspergillus versicolor* and *Stachybotrys chartarum* are also found in the hospital aeromycota [1].

The most important aerobiological parameters affecting mold infections include the size of infectious particles, the temperature and relative humidity of the room, and human activity in the sampled room [18,19]. In addition to the above parameters, the composition of indoor aeromycota also depends on external aeromycota, which vary according to climatic conditions such as precipitation, wind speed and seasons [20,21]. Depending on the size of the infectious particles, we divide infections into those that spread via droplets and those that spread via aerosols—droplet transmission applies to particles larger than 5 µm and aerogenic transmission in cases where the particles are smaller than 5 µm [18]. Due to their size, the latter remain suspended in the air for a long time, travel with the airflow and can enter the lower respiratory tract [18,22]. The conidia of most molds are significantly smaller than 5 µm and can easily travel long distances through the air under favorable conditions [22]. The conidia of all mold species exhibit some degree of hydrophobicity, which affects their aerosolization and dispersal in the environment, but the conidia of *A. fumigatus* are significantly more hydrophobic than the conidia of other species and can, therefore, disperse more successfully in the environment [22]. The small size (2–3 µm in diameter), spherical shape and pronounced surface hydrophobicity of *A. fumigatus* conidia, as well as their efficient adaptation to temperature fluctuations, nutrient deficiency and varying relative humidity, are the main reasons why *A. fumigatus* is the most common and important opportunistic mold in the hospital environment [1,16,22,23].

## 3. Air Sampling

Approximately one fifth of all hospital infections are attributable to the aerogenic route, as are post-operative wound infections [18,24]. Molds represent a special category of difficult-to-control hospital pathogens due to their ubiquitous presence, their ease of aerosolization, their ability to float for long periods of time and their ability to enter indoor environments from outside. Monitoring the “infectivity” of the air is important for certain patient groups and can be performed in different ways: the first way is the qualitative and quantitative assessment of aerosolized particles according to the ISO 14644-1:2015 standard [25], and the second way is the microbiological monitoring of operating theatres [3,26]. In the first case, the methodology is characterized by high reproducibility and accuracy, but the results correlate poorly with the microbial load, making it difficult to assess the risk of healthcare-associated infections [27,28]. When we use microbiological methods, we obtain results that most realistically represent the microbial contamination and, therefore, the risk to the patient. The principles and basic methodology of this biocontamination control are formalized in ISO 14698-1:2003 [26]. It is not yet clear whether there is a significant correlation between particle counting and microbial loads, as the studies provide different results [29,30].

By taking air samples, we are trying to indirectly determine the adequacy of the conditioned and controlled ventilation systems (taking samples in operating theatres at rest) and the teams’ hygiene procedures (taking samples in active, functioning operating theaters) in addition to the direct risk to the patients [3,14,27,31,32]. The relationship between microbiological and dust contamination has been proven, with surgical technique and duration of surgery being the most important predictors [33]. It has been shown that the microbial contamination of the air increases significantly during surgical procedures, with higher levels in the patient area [8,9,34].

Air sampling methods include settle plates, air filtration, liquid- or solid-phase inertial impaction and electrostatic sampling [23]. The sampling of bioaerosols in operating theaters is usually performed using one of these two methods [26,31,35,36]:

- passive sampling, which is based on the settling of microbes on solid media;
- active sampling, in which a known volume of air is physically drawn onto solid or liquid media or into various liquids.

Both methods are intended for the detection of molds and bacteria in the air, while the active sampling version also enables the detection of viruses. The choice of sampling method is the main issue for the standardization process—the two methods differ in almost every aspect, from ease of performance, sensitivity and accuracy to the way the results are presented and interpreted. Many recommendations and standards specify limits for acceptable concentrations of microorganisms in the air, but they do not explicitly state the method of sampling [3,14,26,28,31,36].

The two methods are considered fairly equivalent in certain situations, but we must choose one or the other, as combining the methods can lead to an unclear presentation and interpretation of the results [31,35]. The equivalence is particularly evident in the sampling of rooms in which a higher microbial load is to be expected, while in the sampling of (ultra)clean rooms such as operating theaters and hospital rooms with positive pressure, the active sampling methods take the lead due to their better sensitivity [3,26,27,31,37]. The problem with both methods, as in medical mycology in general, is the distinction between clinically important isolates and contaminants. Apart from strict adherence to aseptic working techniques to reduce the risk of contamination, there are no other methods that clearly define whether an isolate is a contaminant or not. Careful consideration is always recommended before reporting final results.

### 3.1. Passive Sampling Methods

**Figure 1:**
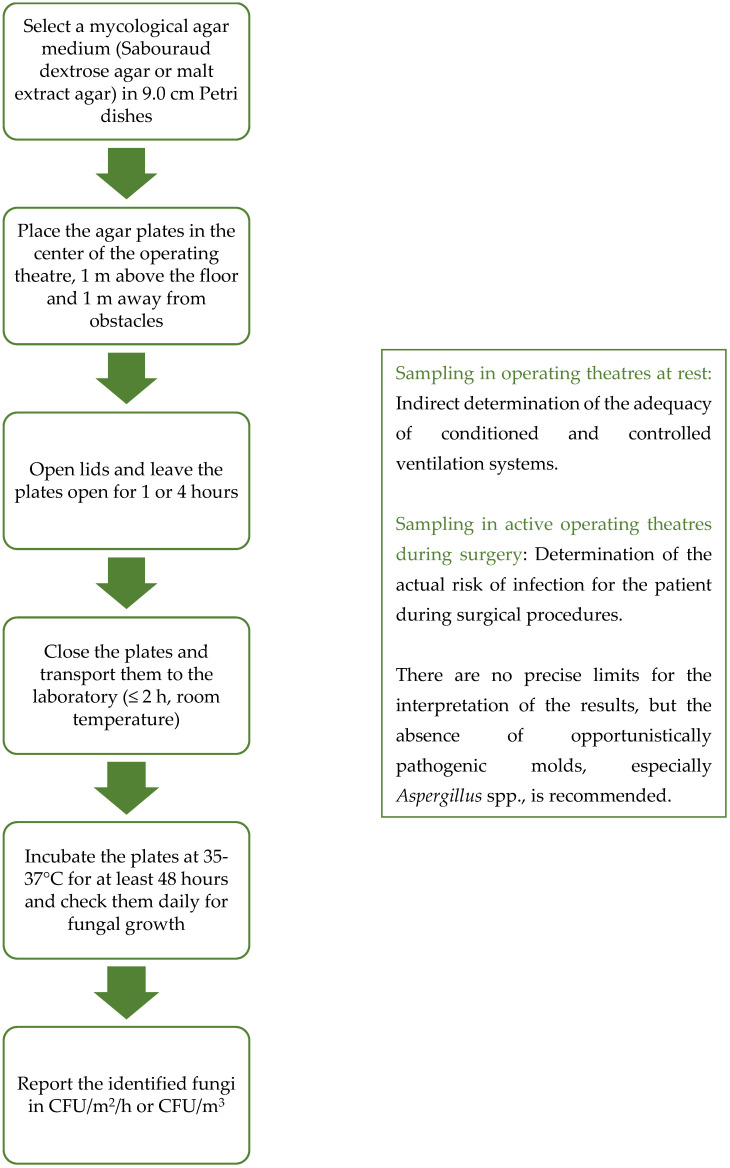
Flowchart of passive air sampling. The diagram shows the main steps of the passive air sampling procedure described in this mini-review article. Certain steps can be adapted, e.g., other agar plates or a different temperature and incubation time can be used if other than opportunistic pathogenic molds are to be cultured. When interpreting the results, it should be borne in mind that passive air sampling is only suitable for sampling ultraclean rooms to a limited extent due to its lower sensitivity and false-negative results.

Passive air sampling methods were first introduced in pharmaceutical production and later transferred to medical facilities [3,35]. Passive air sampling is probably the most readily available and economical method of bioaerosol sampling [38,39]. There is only one method of passive sampling—the sedimentation method—in which open agar plates are exposed in space for a period of time [26]. After exposure, the settle plates are covered and incubated at the selected temperature (Figure 1). As thermotolerant fungi are of particular concern in immunocompromised patients due to their pathogenicity, samples should be incubated at 35–37 °C [3]. There are many variants, of which the 1/1/1 method is the most common and has been used since the 1970s [40]: the agar plates (diameter 9.0 cm) are placed on a stool at a height of 1 m above the floor, keeping a distance of at least 1 m from physical obstacles such as walls, windows and entrance doors or other obstacles in the environment, and sampling should take 1 h [26,30,32]. More than 20 years ago, Pasquarella et al. made a major contribution to the standardization of passive air sampling and the interpretation of results with the Index of Microbial Air Contamination (IMA) [41]: contamination classes and maximum acceptance values for different infection or contamination risks were defined. Five IMA classes were defined, representing different, increasing degrees of contamination: 0–5 very good; 6–25 good; 26–50 fair; 51–75 poor; and >76 very poor [41]. A maximum value of 5 IMA and 25 IMA was recommended in ultraclean and conventional operating theaters (Table 1) [24,41]. The IMA standard has been incorporated into the Swiss national guidelines, which recommend a target value, an alarm value and an action value of 2, 2–5 and 5 IMA, respectively, for arthroplasty and 15, 15–25 and 25 IMA, respectively, for general surgery [24,42]. Using the 1/1/1 method and the IMA is a good way to standardize passive sampling and compare results. A version of the sedimentation method is also described in the European Medicines Agency (EMA) guidelines for good manufacturing practice (EU GMP) and is primarily intended for the pharmaceutical industry, but Pasuqarella et al. [8] managed to identify parallels for use in operating theaters (Table 1) [8,35]. The technical implementation of sampling is only mentioned indirectly, but the interpretation criteria and cut-off values are clearly defined [35]. The method of sampling is constant between the versions of the sedimentation method, the use of culture media and the incubation time; the interpretation of the results mainly vary. Depending on the microorganisms we want to detect in the air, many solid culture media are used [3]. Among the culture media, specific mycological media such as malt extract agar and Sabouraud dextrose agar (with or without antibiotics) are commonly used and recommended, while other non-specific media such as tryptic soy agar do not optimally support fungal growth [3,14,19,43].

The sedimentation method is based on the settling of conidia and sporangiospores on an exposed culture medium, so that the results are expressed in the settling rate in colony-forming units per square meter per hour (CFU/m^2^/h) [3,36,41]. The results can be given in many different ways, which makes comparison difficult—in addition to CFU/m^2^/h, results can also be expressed as CFU/dm^2^/h, CFU/cm^2^/h, CFU/plate or even CFU/m^3^ if using Omelyansky’s formula [3,36,39,44]. The sedimentation rate of bacteria is much higher than that of molds because they can float and move in the air for long periods of time. Therefore, the sensitivity of the sedimentation method for the detection of bacteria is also better, at least when sampling in cleanrooms [18,19,22]. Some recommendations, therefore, advise against using the sedimentation method to determine the concentration of molds in the air [3,45].

Most recommendations and guidelines for the microbiological control of the hospital environment, especially in operating theatres, do not specify limit values for sampling when using the sedimentation method, but only specify limits in CFU/m^3^ when using active sampling methods [14,31,41]. The interpretation of the results is best standardized by the IMA and the EMA EU GMP, both of which specify limit values for sedimentation methods for rooms with different degrees of cleanliness [35,41]. The first is intended for all types of indoor environments, including hospitals, and the second only for the pharmaceutical industry, although they can also be used to some extent to control operating theater environments [8]. For example, most operating theaters can be classified as Categories A or B rooms when they are not in use and Category C rooms when they are active [8]. The main methodological difference between the IMA 1/1/1 method and the EMA EU GMP is the exposure time: 1 h for the first and 4 h for the second method. The difference also lies in the interpretation of the results. In contrast to the IMA, the EMA EU GMP explicitly sets limits for fungi, 0 CFU/plate for Categories A and B rooms, while the IMA proposes a limit for the total number of microorganisms (bacteria and fungi) of up to 9 CFU/m^2^/h for high-risk rooms [35,41]. Similarly, in Switzerland, limits have been set for operating theatres with turbulent airflow, namely, up to 786.4 CFU/m^2^/h (bacteria together with molds) for operating theatres at rest and up to 3932.1 CFU/m^2^/h (bacteria together with molds) for active operating theatres [14,24,42]. The limits are usually set for both bacteria and fungi together, but the authors agree that pathogenic fungi, especially *Aspergillus* spp., should not be present [31,43].

The main advantages of the sedimentation method are definitely the ease of implementation, the fact that the natural air flow is not disturbed and the results correlate with the actual risk (the microorganisms obtained on agar plates are the ones that can actually cause infections by colonizing/infecting anatomically sterile areas) [41]. The disadvantages include the relatively low sensitivity in operating theaters and the lack of limit values in international guidelines [3,41].

### 3.2. Active Sampling Methods

Active air sampling is performed using volumetric methods in which a known volume of air is physically drawn onto solid or liquid media or into various liquids or onto polycarbonate membrane filters [38]. It has been shown that volumetric methods are more sensitive than sedimentation methods in ultraclean rooms, as they also detect microorganisms with a low sedimentation rate [14,45].

**Figure 2:**
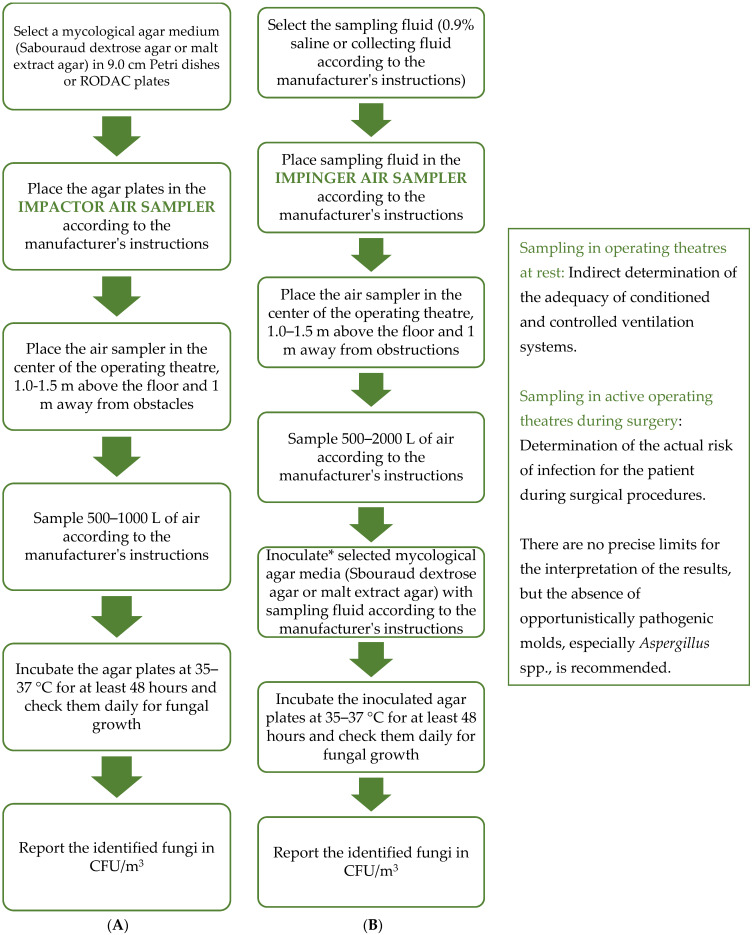
Flowchart of active air sampling. The diagram shows the main steps of the impactor (**A**) and impinger (**B**) air sampling procedures described in this mini-review article. Certain steps can be adapted, e.g., other agar plates or a different temperature and incubation time can be used if other than opportunistic pathogenic molds are to be cultured. Many different air samplers are commercially available, so it is very important to follow the manufacturer’s specific instructions. * Instead of traditional cultivation, the impinger method of sampling also enables molecular and chemical analyses of the air.

Roughly speaking, a distinction can be made between impactor and impinger sampling. In impactor sampling, the air is aspirated directly onto the agar plates or polycarbonate filter membranes, whereas in impinger sampling, it is aspirated into a specific liquid that can be used either to inoculate the agar plates or for molecular biology studies [46,47] (Figure 2). Various types of samplers are known for both impactor and impinger sampling, which differ in terms of air flow rate, volume of air collected, number of agar plate/membrane carriers, etc. When collecting air samples, we must be aware that air is a very complex sample whose microbiological composition depends on many factors, which is associated with low reproducibility [3,19,37,47,48]. The amount of air collected is, therefore, of crucial importance for the reporting and interpretation of the results, which depend mainly on the degree of cleanliness of the sampled operating theatre [49]. A greater representativeness of the sample is achieved through a larger volume, so the tendency is to sample as large a volume as possible [49]. In the literature, different volumes are given for the same type of room, which makes it difficult to develop common guidelines. For example, for operating theaters, it is recommended to sample 0.25 to 2.0 m^3^ of air [8,14,27,31,32,49], or there are no such recommendations at all [35]. Meanwhile, 0.5 m^3^ or 1.0 m^3^ are commonly used (Table 2) [8,14,31,32,50]. Volume limitations are also set by the methods themselves—with impactor samplers, we cannot collect samples for more than 10 min or more than 1000 L; otherwise, the culture medium will dehydrate too much [27,49,51]. We do not have this problem with impinger air samplers because we can, at least theoretically, collect more than 10 m^3^ [47]. The main disadvantage of impinger samplers is that a lot of liquid evaporates during sampling, up to 500 µL/min at 20 °C and 60% relative humidity, which leads to the renewed aerosolization of the already sampled microorganisms and, thus, to falsely lower microbial concentrations [47].

When sampling in an operating theatre at rest with an impactor or impinger sampler, the ventilation system must be turned on 15–60 min before the air sampling to ensure optimal results without interference [18,30]. The air sampler should be placed in the central part of the operating theatre, approximately 1.0–1.5 m above the floor, at the level of the operating table [18,30,31]. The choice of the culture media is similar to passive sampling—specific mycological culture media such as Sabouraud agar are recommended [3,14].

The main advantage of volumetric methods is their high sensitivity, which is mentioned in national guidelines, and the established limit values in CFU/m^3^ for different types of operating theatres [3,14,27,31,35]. Although airborne spores of various molds may pose a risk to neutropenic patients, the critical number of these spores above which outbreaks of mycoses would be expected is not defined [3]. Regarding fungal contamination in general, lower levels were found in operating theatres than in other hospital environments, indicating the effectiveness of air conditioning systems in reducing fungal contamination [52]. Despite these results, there are still no generally accepted limit values for fungal concentrations in the air of operating theatres. The limit values vary and are mainly proposed depending on the type of ventilation of the operating theatre and the activity in the room during sampling (operating theatres at rest and active operating theatres) [3,14,31,35,53]. These limit values are formulated for bacteria and fungi together, similar to passive air sampling. Most authors agree that indicator microorganisms should not be present in high-risk operating theaters: species from the genus *Aspergillus*, especially *A. fumigatus*, and species from the order *Mucorales*, which pose the greatest risk to patients [31,35,54].

The main disadvantage of active sampling is the air turbulence during sampling, as this disturbs the natural air flow. As a result, we obtain biased information for risk assessment as we also consider airborne microorganisms that do not pose a direct risk to the patient as they are more difficult to settle [41].

**Table 2:** Some examples of active air sampling for fungi in operating theaters.

Sampling Method	Air Volume	Flow Rate	Mycological Medium	Incubation Time and T	Reference
Impactor air sampler	500 L	180 L/min	SabC	10 days, 30 °C	Napoli et al., 2012 [14]
	500 L	180 L/min	SabC	2 days, 37 °C	Pasquarella et al., 2012 [8]
	283 L	28.3 L/min	SabC	7 days, 37 °C	Abbasi and Samaei 2019 [55]
	1000 L	/	/	/	ISPESL 2009 [31]
	250 L	100 L/min	MEA	5–7 days, 27 °C	Viegas et al., 2020 [56]
	600 L	180 L/min	PCA	/	Squeri et al., 2019 [50]
Impinger air sampler	3000 L	300 L/min	MEA	7 days, 25 °C	Chang et al., 2019 [47]
	1000 L	/	PCA	2 days, 37 °C	Mntagna et al., 2019 [30]
	600 L	300 L/min	/ *	/ *	Viegas et al., 2020 [56]

* Molecular tests. SabC—Sabouraud agar supplemented with chloramphenicol; MEA—Malt extract agar; PCA—Plate count agar.

## 4. Sampling Frequency

There are no international guidelines and recommendations that prescribe the frequency of sampling in individual departments. In addition to the technical and scientific literature, there are also regional and national recommendations [27,31,50,54,57] on this subject. It is, therefore, recommended to take air and surface samples [31,35,50,54,57]:

- Routinely once every six or twelve months;
- After renovation and adaptation work;
- After construction work;
- After changes to the protocols for cleaning and disinfecting ventilation systems;
- After longer periods of non-use of the premises;
- If the limit values are exceeded;
- In the event of a proven fungal infection of the endoprosthesis after surgery.

## 5. Conclusions

Studies have repeatedly found the presence of fungi in the air of operating theaters, with *Aspergillus* being a common genus. The extent of biocontamination in hospitals varies, with higher levels in working operating theaters. The use of air-conditioning systems has been shown to reduce the number of microorganisms, including fungi, in the hospital environment. However, air-conditioning filters can also be a source of fungal contamination. These findings underscore the importance of regular air sampling and maintenance of air-conditioning systems in operating theatres to minimize the risk of fungal infections. Mycological air monitoring is based on active and passive sampling, methods that are particularly comparable in environments with an expected higher microbial load. In operating theaters, active air sampling is the leading method, which is mainly because of its high sensitivity. Within active sampling, we find many methods based on the use of impactor or impinger samplers. The principles of the sampling method are formalized in the ISO 14698-1:2003 standard. The air samples should be taken in the center of the operating theatre, within the operating field, 1.0–1.5 m above the floor. We should take 250 L to 1000 L of air. By taking air samples, we try to indirectly determine the adequacy of the conditioned and controlled ventilation systems (taking samples in operating theatres at rest) and the teams’ hygiene procedures (taking samples in active, functioning operating theaters) in addition to the direct risk to the patients. The use of specific mycological cultures, such as malt extract agar and Sabouraud agar, is recommended for the detection of fungi. The aim is to detect medically important fungi; therefore, an incubation of at least 48 h at 37 °C is recommended. There are still no guidelines and recommendations that would precisely define the advisable concentrations of fungi in the air, but most authors recommend the absence of opportunistic molds, especially of the genus *Aspergillus*. Further research is needed to improve and standardize air sampling protocols and interpretation of results.

## Figures and Tables

**Table 1:** Methods of passive air sampling and interpretation according to IMA and EMA EU GMP (adapted from Pasquarella et al., 2000, 2012 [8,41]).

EMA EU GMP Grade	IMA Places at Risk for Infection	EMA EU GMP Settle Plates (CFU/plate/1 h)	IMA Settle Plates (CFU/plate/1 h)
A	Very high risk *	0	0
B ^§^	High risk ^+^	1.25	5
C ^¶^	High risk	12.5	-

* Operating theatre for joint replacement and other ultraclean rooms. ^+^ Conventional operating theatres and other clean rooms. ^§^ Operating theatres at rest. ^¶^ Active operating theatres (in operation). IMA—Index of microbial air contamination. EMA EU GMP—European Medicines Agency, European Union Good Manufacturing Practice. CFU—Colony Forming Unit.

## Data Availability

No new data were created or analyzed in this study. Data sharing is not applicable to this article.
